# Regulatory Role of CD4^+^ T Cells in Myocarditis

**DOI:** 10.1155/2018/4396351

**Published:** 2018-06-21

**Authors:** Daria Vdovenko, Urs Eriksson

**Affiliations:** ^1^Cardioimmunology, Center for Molecular Cardiology, University of Zurich, Zurich, Switzerland; ^2^Department of Medicine, GZO-Zurich Regional Health Center, Wetzikon, Switzerland

## Abstract

Myocarditis is an important cause of heart failure in young patients. Autoreactive, most often, infection-triggered CD4^+^ T cells were confirmed to be critical for myocarditis induction. Due to a defect in clonal deletion of heart-reactive CD4^+^ T cells in the thymus of mice and humans, significant numbers of heart-specific autoreactive CD4^+^ T cells circulate in the blood. Normally, regulatory T cells maintain peripheral tolerance and prevent spontaneous myocarditis development. In the presence of tissue damage and innate immune activation, however, activated self-antigen-loaded dendritic cells promote CD4^+^ effector T cell expansion and myocarditis. So far, a direct pathogenic role has been described for both activated Th17 and Th1 effector CD4^+^ T cell subsets, though Th1 effector T cell-derived interferon-gamma was shown to limit myocarditis severity and prevent transition to inflammatory dilated cardiomyopathy. Interestingly, recent observations point out that various CD4^+^ T cell subsets demonstrate high plasticity in maintaining immune homeostasis and modulating disease phenotypes in myocarditis. These subsets include Th1 and Th17 effector cells and regulatory T cells, despite the fact that there are still sparse and controversial data on the specific role of FOXP3-expressing Treg in myocarditis. Understanding the specific roles of these T cell populations at different stages of the disease progression might provide a key for the development of successful therapeutic strategies.

## 1. Introduction

Myocarditis represents a polymorphic, frequently infection-triggered, and immune-mediated inflammation of the heart muscle [1]. Most often, it resolves spontaneously, but in susceptible individuals, it can progress to a chronic stage, which finally results in pathological cardiac remodelling. Pathological remodelling includes tissue fibrosis, hypertrophy, and apoptosis of cardiomyocytes and results in a phenotype of dilated heart chambers with impaired contractility (inflammatory dilated cardiomyopathy (iDCM)). Patients with iDCM develop heart failure with high mortality [2]. In children, myocarditis leads to cardiomyopathy in 46% of affected individuals [3], and up to 20% of sudden death cases in young adults have been reported to be due to myocarditis [4]. Diagnostic gold standard is myocardial biopsy, despite a lack of sensitivity, mainly due to sampling error [2, 5]. Nevertheless, appropriate histological, immunohistochemical, and molecular biological workup of sufficient numbers of heart biopsies greatly improved diagnostic accuracy and allows meanwhile not only a morphological classification but also detection of replicating viral genomes in the heart [6, 7].

Viral infections are the most frequent cause of myocarditis along with some bacteria, and protozoa. Moreover, toxins, vaccines, and several drugs, as well as systemic autoimmune diseases, can also trigger heart-specific autoimmunity and inflammation [8]. Following tissue damage of any cause, the release of cardiac self-antigens and activation of scavenging self-antigen-presenting dendritic cells in draining lymph nodes may result in a breakdown of heart-specific tolerance triggering production of heart-specific autoantibodies, autoreactive CD4^+^ T cell expansion, and autoimmunity [9, 10]. Various intracellular cardiac peptides, surface receptors, and mitochondrial antigens had been reported as markers of cardiac injury [11], but not all of them are heart specific or promote autoimmunity. Autoantibodies to both cardiac troponin T and I had been detected in sera of mice and men, but only immunization with troponin I led to myocarditis in mice [12, 13]. Autoantibodies to beta1-adrenoceptors had been shown to promote dilated cardiomyopathy in rodents [14, 15] and are associated with adverse outcome in patients with dilated cardiomyopathy [16, 17] or Chagas heart disease [18]. Patients with dilated cardiomyopathy also demonstrate increased serum levels of autoantibodies to M(2) muscarinic acetylcholine receptor. In mice, adoptive transfer of M(2) muscarinic acetylcholine receptor-specific splenocytes induces myocarditis, with T cell infiltrations in the heart and a dilated cardiomyopathy-like phenotype [19]. Epitopes of the alpha-myosin heavy chain (*α*-MyHC) peptide are heart specific, highly immunogenic in various animal models, and associated with autoantibodies and T cell-mediated myocarditis both in mice and humans [20–23].

CD4^+^ T cells were defined as main drivers of heart-specific autoimmunity in myocarditis [24–27]. Expansion of heart-specific effector CD4^+^ T cells is facilitated in humans and mice due to a high frequency of circulating naïve *α*-MyHC-specific CD4^+^ T cells. The high frequency of *α*-MyHC-specific CD4^+^ T cells is a result of defective negative selection in the thymus. In fact, transcripts of *Myh6*, the gene encoding murine *α*-isoform of myosin heavy chain, are absent in mouse medullary thymic epithelial cells. Humans also do not express *α*-MyHC in mTECs. Accordingly, patients with inflammatory cardiomyopathy demonstrate increased T cell responses against *α*-MyHC [28]. Taken together, a natural gap in negative selection of *α*-MyHC-specific CD4^+^ T cells can explain susceptibility to heart-specific autoimmunity in the context of tissue damage, self-antigen release, or exposure to pathogen-derived molecules mimicking cardiac proteins [29].

Effector CD4^+^ T cells (Teff) were reported to be critical for myocarditis development in patients and animal models [24, 30]. Starting from their naïve form, CD4^+^ T cells differentiate into either mature effector or regulatory cell populations with distinct functions [31, 32]. Aside from CD4^+^ T cell subsets including regulatory T cells (Treg), several other cell types can exert a regulatory suppressive function in myocarditis development. Such cells include bone marrow-derived progenitor cells, CD8^+^ T cells, monocytes/alternatively activated macrophages, or dendritic cells [33–37]. Regulatory T cells, expressing forkhead box P3T (FOXP3), suppress effector cells and maintain immune homeostasis and tolerance in various autoimmune disease models, but their role in myocarditis is still debatable [38–41]. Importantly, there is functional polymorphism and high plasticity in all the different T cell subpopulations [42, 43]. In fact, the regulatory role of the different T cell subtypes in myocarditis highly depends on the stage of disease and on a complex and not yet understood interaction between different inflammatory heart infiltrating and heart resident cell types. IFN-*γ*-producing Th1 effector T cells can convert to suppressor cells [44]. Vice versa, Treg are also able to produce proinflammatory cytokines under certain conditions [45]. In fact, dual IL-17-producing FOXP3^+^ regulatory T cells may play a critical role in controlling inflammatory balance in humans [46]. Whether these observations are also valid in the context of cardiac inflammatory diseases is not known, however. In this review, we will focus specifically on the regulatory role of different CD4^+^ T cell subtypes in general in the context of myocarditis and its progression to inflammatory dilated cardiomyopathy. Our current knowledge largely bases on mouse and rat models of viral and experimental myocarditis, as well as from observational studies on patients with myocarditis or inflammatory dilated cardiomyopathy.

## 2. CD4^+^ T Cells as Critical Mediators of Heart-Specific Autoimmunity

Autoimmune mechanisms play an important role in myocarditis development and in its progression to inflammatory dilated cardiomyopathy. In patients and mice with myocarditis, heart-specific autoantibodies can be detected [5, 11]. The role of these autoantibodies for disease induction and progression, however, is still largely speculative [47]. In patients with acute myocarditis, biopsies demonstrate accumulation of T cells and macrophages, as well as other inflammatory cells in close contact to injured cardiomyocytes [48, 49]. Many studies, most of them based on mouse models, indicate an exclusive role for CD4^+^ T cells in myocarditis development and progression. Susceptible mouse strains develop myocarditis after viral, especially coxsackievirus B3 (CVB3), infection [50], as well as upon injections of *α*-MyHC peptide together with complete Freund's adjuvant [51] or activated *in vitro α*-MyHC-loaded bone marrow-derived dendritic cells [9]. Transgenic mice carrying a CD4^+^ T cell receptor specific to cardiac myosin spontaneously develop myocarditis progressing to lethal-dilated cardiomyopathy [52]. In all of these mouse models, myocarditis is associated with a marked *α*-MyHC-reactive effector T helper (Th) cell response. These cells are directly pathogenic, because adoptive transfer of heart-specific CD4^+^ T cells can induce myocarditis in irradiated recipients, SCID, or Rag2^−/−^ mice [24].

## 3. T Cell Maturation: Where Is the Breach?

**Figure 1:**
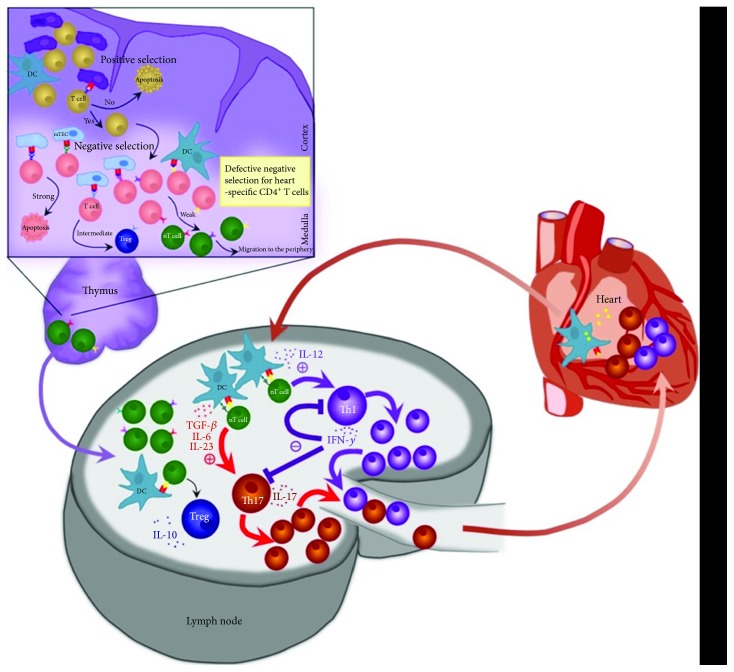
Role of CD4^+^ T cells in myocarditis. Break of central tolerance: CD4^+^ T cells undergo maturation and selection in the thymus. Due to a defect in negative selection, *α*-MyHC-specific CD4^+^ T cells do not undergo anergy or apoptosis and are released to the periphery. Break of peripheral tolerance: Inflammation results in activation of *α*-MyHC-loaded DCs which upregulate MHC II-peptide complexes as well as costimulatory molecules on the surface and migrate to the draining lymph nodes, where they interact with circulating T cells. Activated through the TCR meeting cognate peptide and upon costimulation with CD28, naive heart-specific T cells differentiate to effector T cells entering the heart.

Random recombination in the generation of the diversity of the T cell receptor (TCR) repertoire is an important evolutionary mechanism allowing T cells to specifically recognize and eliminate a large variety of foreign antigens. However, it also harbours potential danger of generating self-reactive clones. Under normal healthy conditions, there are two distinct stages of central and peripheral tolerance, which prevent autoimmunity during the development and activation of T cells. Central tolerance is based on clonal deletion and clonal diversion and is responsible for extracting self-reactive lymphocytes in the thymus [53]. Positively selected for their ability to recognise MHC complexes, thymocytes migrate to the thymic medulla and undergo a process of negative selection. Bearing strongly self-reactive TCRs, cells respond to self-peptide-MHC complexes on medullary thymic epithelial cells and receive apoptotic signals. The autoimmune regulator (AIRE) protein has been shown to play a major role in expression of self-tissue-specific epitopes in these complexes [54]. Humans and mice with compromised or absent AIRE suffer from variable severe autoimmunity in almost all their organs [55]. Just recently, Fezf2, another transcription factor, was introduced to directly regulate various tissue-restricted antigen genes in mTECs independent of AIRE [56]. The key mechanisms however are still largely unknown. Meaningfully, and as mentioned above, the expression of *α*-MyHC is missing in both, humans and mouse mTECs, leading to a defect in negative selection of alpha-myosin heavy chain-specific CD4^+^ T cells in the thymus putting them at risk for autoimmune myocarditis development in the presence of self-antigen release and innate immune activation (Figure 1).

CD45^+^MHCII^+^ bone marrow-derived antigen-presenting cells (APC) constantly process heart-specific epitopes in the heart [57]. This observation was made in many rodent strains, including some, which are not susceptible to viral or immune-mediated myocarditis. Obviously, presentation of cardiac antigens alone is not sufficient for activation and expansion of Teff and myocarditis development. Activation of CD4^+^ T cells requires not only interaction with a cognate antigen expressed on the MHC class II molecule but also costimulatory signals, such as those mediated by CD28 ligation [58]. In the absence of a local inflammatory milieu, DCs do not express sufficient amounts of costimulatory B7 family molecules and are supposed to play a tolerogenic role. CD4^+^ T cells interacting with MHC peptide without costimulatory signal undergo anergy, repression of TCR signalling, and IL-2 production [59].

Surface APC molecules programmed death 1 receptor (PD-1) and cytotoxic T-lymphocyte associated protein 4 (CTLA-4) play an important role in T cell anergy. Deficiency in PD-1 or CTLA-4 leads to impaired peripheral tolerance and enhanced T cell activation [60]. In fact, mice deficient for CTLA-4, PD-1, or its ligand demonstrate elevated numbers of effector T cells and develop severe autoimmune myocarditis and DCM [61–64]. By maintaining signalling through these molecules, DCs mediate the peripheral conversion of naive T cells to Treg. Acute inflammatory processes in the heart, on the other hand, result in upregulation of MHC II peptide complexes as well as costimulatory molecules on the surface of DCs and enhance migration of DC to the draining lymph nodes, where they interact with circulating T cells. This leads to a breakdown of peripheral tolerance and differentiation of naïve T cells into an effector phenotype (Figure 1).

## 4. The Role of Treg in Myocarditis

Since first identified and described as suppressive “regulatory” T cells [65], Treg were intensively studied [66]. Regulatory CD4^+^CD25^+^ T cells represent a specific T cell population responsible for immune homeostasis and tolerance. Their frequencies in the circulation can widely differ depending on the conditions or stage of disease [67]. Treg express FOXP3 transcription factor, which is essential for active suppression of autoimmunity [68]. As other T cells, Treg mainly develop in the thymus, but can also develop in the periphery. Treg suppress autoimmune Teff populations as well as APCs involved in priming and activation via different cell-cell contact-dependent and contact-independent mechanisms. Treg produce inhibitory cytokines such as transforming growth factor beta (TGF-*β*) and IL-10 or express surface molecules with immunosuppressive properties such as CTLA-4 or glucocorticoid-induced tumor necrosis factor receptor (GITR) modulating immune processes [69–71]. Expansion of regulatory cells is an important mechanism to control autoimmunity. In mouse and rat models of experimental autoimmune myocarditis, EAM numbers of Treg conversely correlated with disease severity. Moreover, the proliferation capacity and inhibitory activity of Treg increased in animals immunized for EAM induction [72, 73]. Adoptive transfer of CD4^+^ T cells depleted from highly efficient glucocorticoid-induced TNFR family-related gene/protein-expressing Treg resulted in more severe myocarditis in T cell-deficient BALB/c nude mice [74]. Furthermore, adoptive transfer of Treg protected mice from CVB3-induced myocarditis [75] and from progression to cardiomyopathy, if injected after clearance of the acute virus infection [76, 77].

Differences in numbers of circulating Treg explain variations in the susceptibility of different mouse strains to EAM. Comparison of A.SW and B10.S mouse strains sharing the same MHC haplotype showed that development of severe disease in A.SW mice correlated with a lower relative frequency of Treg among the total CD4^+^ T cell count, compared to resistant B10.S animals [26]. Moreover, gender differences in myocarditis development were linked to differences in circulating Treg. Mice with increased estradiol levels, for example, increased numbers of Treg upon immunization and are less susceptible to CVB3-induced myocarditis [78]. Monocytic myeloid-derived cells from female but not male mice promoted expansion of CD4^+^IL-10^+^ Treg [36]. Furthermore, IL-10 producing Treg transferred to immunized Lewis rats efficiently suppressed myocarditis induction [79]. A decrease in IL-10 production and Treg numbers was also observed in *α*-MyHC/CFA-immunized mice after endothelin receptor blockade and resulted in exacerbated EAM [80]. IL-10 efficiently drives the generation of Treg [81] while its immunosuppressive effect includes decreasing MHC II complexes and B7 family costimulatory molecules on the APC surface [82–84]. IL-37 mediated activation of Treg, and IL-10 production downregulates the expression of Th17-related cytokines IL-6 and IL-17 and ameliorates CVB3-induced viral myocarditis [85]. IL-35, on the other hand, was shown not only to have suppressive activities [86] but also to convert naive T cells into a regulatory phenotype [87].

TGF-*β* directly suppresses self-reactive cells, as shown in models of experimental mouse colitis [88] and encephalitis [89], and protects mice against coxsackievirus-induced myocarditis [75]. Moreover, TGF-*β* launches a paracrine positive feedback loop converting naïve into regulatory CD4^+^ T cells [90]. TGF-*β*, however, was shown to promote disease and adverse cardiac remodelling during later stages of myocarditis: TGF-*β*-mediated Wnt secretion promoted myofibroblast differentiation and myocardial fibrosis in EAM [91], while treatments targeting TGF-*β* prevented fibrosis and heart failure [92–94].

Human CTLA4 haploinsufficiency results in serious dysregulation in T and B lymphocyte homeostasis and specifically affects FOXP3^+^ Treg cells [95]. CTLA-4 as a high-affinity receptor interacts with CD80/CD86 signalling [96], causes elimination of these molecules via transendocytosis [97], and suppresses IL-2—a major T cell survival and expansion factor [98–100]. Adenovirus vector-mediated CTLA4Ig gene transfer in mice with EAM leads to downregulation of CTLA-4 and B7-2 proteins but upregulation of Treg, expression of FOXP3 and TGF-*β* mRNA, and alleviation of myocarditis [73]. Patients with Chagas heart disease demonstrate increased frequencies of suppressive IL-6^+^, IFN-*γ*^+^, TNF-*α*^+^, and CTLA-4^+^ Treg cells but a rather small FOXP3^+^CTLA-4^+^ Treg cell population [101, 102]. Reduction of CTLA-4 levels in CD4^+^ T cells following disruption of T cell Ig mucin signalling during the innate immune response results in decreased Treg populations and increased inflammation in the heart [103]. A direct cytolytic effect of Treg is due to a granzyme B-dependent, perforin-independent mechanism [104] which allows them to eliminate target effector cells.

Interestingly, some observations demonstrate that early activation of Treg might be associated with exacerbation of CVB3-induced myocarditis [105]. Other viral myocarditis models, however, demonstrate the ability of Treg to decrease virus-induced inflammation and to limit tissue damage associated with viral infection [106]. Thrombospondin-2, for example, protected against cardiac dysfunction in acute CVB3-induced viral myocarditis via activation of anti-inflammatory Treg [107]. Valproic acid was suggested as a promising drug in the therapy of viral myocarditis increasing the percentage of Treg cells and decreasing the percentage of splenic Th17 [108]. Moreover, an approach modulating Th17/Treg immune responses by inhibition of microRNA-155 resulted in a simultaneous decrease of both Th17 and Treg and reduced disease severity. These observations, however, suggest that improvement of EAM mainly resulted from the repressed Th17 response [109]. In Chagas myocarditis, granulocyte colony-stimulating factor administration promoted Treg recruitment and reduced cardiac inflammation and fibrosis [110]. In contrast, endogenous administration of CD4^+^CD25^+^ regulatory T cells during *Trypanosoma cruzi* infection was not at all protective in another study. Depletion of Treg via anti-CD25 monoclonal antibodies neither worsened nor improved the outcome of *Trypanosoma cruzi* infection [111].

Attenuation of acute cardiac inflammation by Treg seems to prevent progression of myocarditis to iDCM in humans [112, 113]. Patients with low responder T cell susceptibility to the suppressive function of regulatory T cells demonstrated progression of DCM [114], and an increase of Treg frequency after immunoadsorption therapy improved cardiac function in iDCM patients [115]. In modulating inflammatory responses and inhibiting proinflammatory cytokines, Treg also ameliorate adverse cardiac remodelling after myocardial infarction [116, 117]. Decreased frequencies of circulating Treg in patients negatively correlate with proinflammatory cytokines, such as IL-6, and are associated with a significantly higher incidence of recurrent hospitalization for worsening heart failure [118]. In addition, cell therapy with regulatory T cells prevents chronic rejection of heart allografts in a mouse model of mixed chimerism [119] and enhances mesenchymal stem cell survival and proliferation upon cotransplantation into ischemic myocardium in Yorkshire pigs [120].

## 5. Regulatory Role of CD4^+^ T Effector Cells in Progression of Myocarditis to iDCM

Several observations support a role for CD4^+^ T cells as major drivers of autoimmune myocarditis development [72, 121]. During myocarditis induction, various inflammatory cell subsets infiltrate the heart and produce proinflammatory cytokines, which create an amplification loop enhancing disease progression [72]. The crucial role of self-reactive СD4^+^ T cells in myocarditis induction is well described [10], although mechanisms remain still poorly understood. It is established that IL-17-producing Th17 cells play a major role in initiation and development of myocarditis [122]. Though both Th1 and Th17 cooperate in disease progression and transition to iDCM [52], it was claimed that IFN-*γ* and IL-17 have antagonistic functions in myocarditis and inflammatory cardiomyopathy. Immunosuppressive strategies are beneficial for some patients with iDCM and myocarditis, without evidence on actively replicating viruses in heart biopsies [2]. Thus, elimination of Teff and their proinflammatory cytokines appears as a promising therapeutic strategy. Nevertheless, some contradictory findings have also been reported. It was shown recently that T cells—Treg, Th1, and Th17 in particular—possess great capacity to plasticity and are able to change their function and phenotype depending on the local milieu in tissue and lymph nodes. CD4^+^ T cells often coexpress more than one specific cytokine [123]. Th17 cells, for example, often produce IL-17 and IFN-*γ* [124]. In fact, in a model of experimental autoimmune encephalomyelitis (EAE), it was shown that IL-23-induced IL-17-producing Th17 demonstrated plasticity, that is, the capacity to change their cytokine production profiles in different inflammatory settings. Using a reporter mouse strain designed to fate map cells that have activated IL-17A, Hirota et al. demonstrated that former Th17 cells produced almost exclusively IFN-*γ* and other proinflammatory cytokines in the spinal cord [125]. Another study of effector cell plasticity underlines the nonstability of the IL-17^+^/IFN-*γ*^+^ population and further differentiation to IL-17 or IFN-*γ* single-producing cells [126]. Both Th1 and Th17 undergo active expansion in autoimmune myocarditis, and the balance between these populations may strongly influence disease phenotype and outcome. It was observed that *α*-MyHC/CFA-immunized IFN-*γ*- and IFN-*γ*R-deficient mice develop more severe and persistent myocarditis [127, 128], suggesting a protective regulatory role of IFN-*γ* in this disease model. While in wild-type mice inflammatory infiltrates largely subside within few days after the peak of disease, IFN-*γ*R-deficient show ongoing expansion of autoreactive CD4^+^ T cells, persistent inflammatory infiltrates, and enlarged, functionally impaired hearts with impaired nitric oxide production [128]. It was then confirmed that IFN-*γ* signalling is crucial for NO production by inducible nitric oxide synthase (NOS) 2 in tumor necrosis factor-*α* and NOS2-producing dendritic cells, which limit expansion of Teff and cardiac inflammation [33]. In fact, the progressive disease course in IFN-*γ*R-deficient mice was associated with enhanced IL-17 release from heart-infiltrating Th17 cells. The EAM model also demonstrated that IL-17 recruits CD11b^+^ monocytes confining disease progression in an IFN-*γ*-dependent manner [129]. Moreover, IFN-*γ* signalling was crucial for prevention of EAM by vaccination of mice with FMS-like tyrosine kinase 3 ligand pretreated, *α*-MyHC-loaded splenic CD8*α*^+^ DCs. In this experimental approach, DC vaccination enhanced the Th1 response, which was considered to negatively regulate expansion of Th17 effector cell expansion [130]. In line with these findings, IFN-*γ*-deficient mice also showed severely impaired systolic and diastolic functions and heart failure [131].

In a mouse model of adenovirus 1 infection-mediated myocarditis, depletion of IFN-*γ* during the acute phase of disease did not affect viral replication, but reduced cardiac inflammation protecting from remodeling and hypertrophy [132]. High IFN-*γ* levels correlated with cardiac damage and dysfunction in an autoimmune myocarditis model enhanced by purinergic receptor P2X7 deficiency [133]. Mice lacking Regnase-1 and Roquin, RNA-binding proteins that are essential for degradation of inflammatory mRNAs, demonstrated increased expression of IFN-*γ*, but not IL-17, and suffered from severe inflammation and fibrosis in their hearts [134]. Dampening IFN-*γ* overexpression by Ebi3, a compartment of IL-27, prevented *T. cruzi-*induced myocarditis in mice [135]. Thus, although some studies indicate a protective role of IFN-*γ* as a negative regulator of Teff responses, the same cytokine can also contribute to myocardial inflammation and pathological remodeling.

Recent findings indeed suggest that Teff may play a dual role in myocarditis progression. IL-17 increases myocarditis severity during the acute inflammatory stage [31, 136]. In contrast, it was observed in a *T. cruzi* infection model that anti-mouse IL-17 antibody increased myocarditis severity and mortality [137]. IL-17 signalling via IL-17RA mediated recruitment of IL-10-producing neutrophils, which in turn protect from the development of fatal cardiomyopathy in this model [138]. In line with these findings, it has been shown that in human Chagas disease patients, low frequencies of IL-17-producing T cells correlate with more severe symptoms and cardiac dysfunction [139]. A link between Th17 and Treg has also been shown in a model of viral myocarditis. Neutralization of IL-17 in mice, with an anti-mouse IL-17Ab, resulted in a decrease in Treg counts and T reg cytokines (TGF-*β*, IL-10) [140]. In patients with inflammatory dilated cardiomyopathy, IL-17 seems essential for the transition of myocarditis to iDCM, but serum levels of IL-17 normalize within one year after the diagnosis, whereas cytokines like IL-6 and TGF-*β* remain permanently increased in these patients [141, 142]. Moreover, low serum concentrations of IL-17 were associated with a worse prognosis for patients after acute myocardial infarction [143].

Mice immunized with pcDNA3-hM2, a DNA plasmid carrying the entire muscarinic acetylcholine receptor M2 (M2AChR) cDNA sequence, develop anti-M2AChR-associated DCM mimicking the human cardiomyopathy phenotype. In this DCM model, mice lacking P2×7 receptors produced lower amounts of IL-17 and higher amounts of IFN-*γ* and showed more severe cardiac dysfunction at later stages of disease [133]. Finally, it was shown that mice lacking both cytokines, IL-17 and IFN-*γ*, simultaneously developed rapidly fatal EAM [144]. In line with these findings, unpublished observations from a group also point to a different role of IFN-*γ* and IL-17 in the development of cardiac fibrosis following acute myocarditis.

## 6. Outlook

Myocarditis development and its progression to iDCM are a very complex process. CD4^+^ T cells are key players in the maintenance of peripheral tolerance, are critical for disease induction, are involved in the progression of acute inflammation to a chronic process of pathological remodelling, and may be part of negative feedback loops confining unlimited heart-specific autoreactive T cell expansion. So far, the delicate interplay between distinct CD4^+^ T cell subsets such as Treg, Th1, and Th17 cells has only been partly deciphered.

Further studies in animal models, as well as in human tissue samples, will be required to fully understand the specific role of all different CD4^+^ T cell subsets in myocarditis. Nevertheless, these mechanistic insights are a critical requirement for the development of novel therapeutic concepts and vaccination strategies.

## Abbreviations

AIRE: Autoimmune regulator
APC: Antigen-presenting cells
cDNA: Complementary DNA
CFA: Complete Freund's adjuvant
CTLA-4: Cytotoxic T lymphocyte antigen-4
CVB3: Coxsackievirus B3
DC: Dendritic cells
EAE: Experimental autoimmune encephalomyelitis
EAM: Experimental autoimmune myocarditis
FOXP3: Forkhead box P3
GITR: Glucocorticoid-induced tumor necrosis factor receptor
(i) DCM: (Inflammatory) dilated cardiomyopathy
IFN-*γ*: Interferon-*γ*
IL: Interleukin
ILR: Interleukin receptor
M2AChR: Muscarinic acetylcholine receptor M2
MHC II: Major histocompatibility complex II
mTEC: Medullary thymic epithelial cells
*α*-MyHC: Alpha-myosin heavy chain
NO: Nitric oxide
NOS: Nitric oxide synthase
PD-1: Programmed death 1 receptor
TCR: T cell receptor
Teff: Effector T cells
TGF-*β*: Transforming growth factor beta
Th: T helper cells
TNF: Tumor necrosis factor
Treg: Regulatory T cells.
